# Fatty Acid Profiles of Raw and Cooked Meat Obtained from the Barents Sea Red King Crab

**DOI:** 10.3390/ani16040651

**Published:** 2026-02-18

**Authors:** Alexander G. Dvoretsky, Vladimir G. Dvoretsky, Fatima A. Bichkaeva, Nina F. Baranova, Olga S. Vlasova

**Affiliations:** 1 Murmansk Marine Biological Institute of the Russian Academy of Sciences (MMBI RAS), 183038 Murmansk, Russia; 2 N. Laverov Federal Center for Integrated Arctic Research of the Ural Branch of the Russian Academy of Sciences (FECIAR UrB RAS), 163000 Arkhangelsk, Russia

**Keywords:** fatty acids, red king crab, raw meat, cooked meat, comparisons, nutrition indices, Barents Sea

## Abstract

Previous studies have reported the fatty acid composition of red king crab (*Paralithodes camtschaticus*) meat; however, no comparative analyses have simultaneously investigated raw (RM) and cooked meat (CM). This study addresses this gap by employing gas–liquid chromatography to analyze the fatty acid profiles of RM and CM from red king crabs from the coastal Barents Sea, Russia. Both products exhibited significant sex-specific differences, with females containing higher levels of fatty acids in their meat than males. Furthermore, males displayed greater dissimilarity between RM and CM compared to females. While nutritional indices were comparable between male and female RM and CM, the cooking process resulted in improved nutritional indices in CM relative to RM. Moreover, the red king crab meat exhibited superior nutritional indices compared to those reported for other, primarily southern, crab species, reflecting a higher polyunsaturated to saturated fatty acid ratio in both RM and CM. Comparisons with the existing literature revealed variability in red king crab meat fatty acid levels across geographical locations and years, suggesting that spatial variations in diet and temporal fluctuations in feeding habits may influence meat quality. Our findings confirm the high nutritional value of red king crab meat, a finding of relevance to both scientific and consumer audiences.

## Simple Summary

This study explores the fatty acid composition of red king crab meat from the Barents Sea. Male crabs exhibit lower fatty acid levels than females, likely due to physiological, behavioral, or activity-related factors. Cooking impacts the meat differently depending on crab sex, with males showing a significant improvement in fatty acid content after cooking, and females displaying less pronounced but still significant changes. These variations may be due to differences in tissue structure between sexes. Although overall fatty acid levels in red king crab meat are lower than in other crabs, the product generally maintains a more balanced ratio of polyunsaturated fatty acids to saturated fatty acids, resulting in favorable nutritional indices. Consequently, red king crab meat emerges as a highly nutritious option suitable for a healthy diet.

## 1. Introduction

The global demand for safe, high-quality protein sources is escalating in response to increasing human population numbers [1,2,3]. Aquatic organisms, including fish and other edible aquatic species, are recognized for their superior nutritional value and widespread consumption as a high-quality food source [4,5]. Within this group, crustaceans hold significant commercial value, commanding premium prices in both domestic and international markets [6,7]. Crabs represent an important component of global food sector in terms of economic value and market demand [8,9], ranking third in global crustacean production, after shrimps and lobsters [10]. The unique textural properties, distinctive taste, and flavor profile of crab meat contribute to its popularity as a premium seafood item in restaurants worldwide [11,12]. Furthermore, crab meat possesses notable nutritional benefits, characterized by high concentrations of digestible protein, essential amino acids, free amino acids, and essential fatty acids [10,13].

The Barents Sea is one of the world’s most productive ocean regions [14,15], featuring large fish stocks and promising aquaculture potential, with large fish stocks [16,17,18]. However, it lacks native commercially important crab species [19,20]. To create a new fishery resource, the red king crab (*Paralithodes camtschaticus*) was introduced in the 1960s [19]. This species quickly established a self-sustaining population in the coastal area. Since 1994, Russian and Norwegian specialists have monitored this population, developing a management regime that led to commercially viable abundances [19]. Consequently, commercial fisheries for the red king crab were opened in Norwegian waters in 2002 and in Russian waters in 2004 [21,22].

The introduction of the red king into the Barents Sea has established it as a significant ecosystem component, stimulating considerable scientific interest in its biology and ecology [19]. Research efforts have encompassed its distribution and migratory patterns [23,24,25,26], molting and growth dynamics [27,28], feeding habits and trophic interactions [29,30,31,32], behavioral ecology [33], reproductive biology [34,35,36], and symbiotic relationships [21,37].

The primary motivation for fishing red king crabs is their meat, which is considered a delicacy due to its high content of amino acids, such as tyrosine, histidine, arginine, tryptophan, and cysteine, as well as its low cholesterol levels [38]. The market value of red king crab meat ranges from $175 to $220 USD, driven by its appealing attributes and fluctuations in crab stocks and landings in native areas, leading to a supply deficit in global markets [39,40]. In the Barents Sea, biochemical studies conducted in recent years have provided new insights into the hormonal regulation of basic physiological processes of red king crabs and the nutritional quality of the crab meat and by-products [41,42,43,44]. Specifically, studies have reported variations in fatty acids in the raw leg meat of red king crabs in terms of both absolute and percentage content [41]. Norwegian authors have also studied the fatty acid content in the cooked meat, reporting data on the percentage content [42] and absolute content [44] for adult male crabs, as well as absolute content in juvenile male crabs [44].

Despite ongoing research, significant knowledge gaps continue to exist regarding the fatty acid composition of red king crabs in the context of human nutrition and food science applications. Notably, no comparative studies have been conducted that simultaneously analyze raw and cooked meat from the same specimens. Such comparisons are essential to determine whether consumers are receiving the expected polyunsaturated fats or if thermal processing results in degradation of these nutrients. Additionally, comparative analyses of fatty acid composition between male and female crabs are limited [41], obscuring potential sex-specific responses to cooking. Furthermore, nutrition quality indices, such as atherogenicity and thrombogenicity indices, based on the fatty acid composition of red king crabs from Russian Barents Sea waters, have yet to be reported, thereby hindering evidence-based dietary recommendations for this commercially significant species.

The aim of the present study was to compare the fatty acid profiles of raw and cooked meat of red king crabs from the Barents Sea and report nutritional indices of these products.

## 2. Materials and Methods

### 2.1. Collection Procedures

Red king crabs were collected in Dalnezelenetskaya Bay, located on the eastern Kola Peninsula coast [45,46], during underwater surveys employing standardized diving transects at depths ranging from 6 to 43 m in July 2021. Once the crabs were collected, they were transported to a coastal laboratory, where they underwent examination for sex, biometric parameters, and weight.

The sex of each crab was visually identified based on the morphology of the abdomen. Measurements of size were obtained using vernier calipers, measuring carapace length as the greatest straight-line distance from the posterior margin of the right eye orbit to the medial posterior margin of the carapace [47]. Crab weights were determined with an electronic balance. All collected crabs were mature specimens, with carapace lengths exceeding 90 mm [28,35].

Following the biometric assessments, specimens (n = 84) were processed by labeling one leg on the right side of each crab. Crabs were euthanized using a mechanical method: a scalpel was employed to swiftly sever the central nervous system. The crabs were then divided into two clusters, each consisting of three walking legs and one claw assembled at the shoulder joint. Raw meat samples were obtained from the merus of the third walking leg by carefully removing the shell with scissors, yielding samples weighing between 10 and 30 g. The opposing cluster was rinsed with cooled running water for 15 min to eliminate free body fluids. Subsequently, the clusters were drained for 30 min and then placed in an enameled pan to be cooked by immersing them in water at 95 °C for 15 min. Afterwards, the clusters were cooled in cold water containing 3.5% NaCl for 30 min. Meat samples from the cooked clusters were also taken from the merus. The boiling method used in our study (salt water, 15 min) reflects standard consumer cooking practices, making our results directly applicable to the fatty acid composition of cooked crabs as typically consumed. Both raw and cooked meat samples were placed in labeled plastic bags and frozen. These frozen samples were then delivered to the laboratory at the Institute of Physiology of Natural Adaptations (Federal Center for Integrated Arctic Research, Arkhangelsk) for fatty acid analysis.

### 2.2. Fatty Acid Assay

Lipid extraction was performed using a modified Folch et al. method [48]. A 0.3–0.5 g homogenized sample was added to a 100 mL glass flask containing 10 mL of a chloroform:methanol (2:1) extraction mixture and a chloroform solution of nonadecanoic acid (C19:0) (0.2 mg C19:0). The mixture was homogenized for 30 min and incubated at 25 °C for 10–12 h for lipid extraction. The solution was filtered, and additional chloroform:methanol was added to achieve a final volume of 15 mL. Three mL of a 0.74% aqueous CaCl_2_ solution was added, and the mixture was refrigerated for 12 h to allow phase separation. The upper, aqueous phase (containing water-soluble impurities) was removed via Pasteur pipette, and the lower, chloroform phase was retained for further analysis. After adding 0.5–1.0 mL methanol, the chloroform phase was evaporated to dryness under vacuum. The residue was dissolved in 0.2 mL of chloroform:methanol and mixed for 5 min. Two mL of a 1.5% solution of H_2_SO_4_ in methanol was added, and the mixture was incubated at 90 °C for 30 min in a water bath. The sample was transferred to a 10 mL tube, 0.8 mL of distilled water was added, and the mixture was incubated at ambient temperature for 2–4 h to allow phase separation. The upper phase was transferred to a 2 mL vial and evaporated under vacuum. Finally, 200 µL of the sample was transferred to a gas chromatography vial for analysis. Fatty acid methyl esters were analyzed using an Agilent 7890A gas chromatograph (Agilent Technologies, Santa Clara, CA, USA) equipped with a flame ionization detector. Separation of the esters was achieved using a 60 m × 0.25 mm × 0.15 μm capillary column, Agilent DB-23. Nitrogen served as the carrier gas at a flow rate of 1 mL min^−1^. Both the injection port and the flame ionization detector were maintained at a temperature of 250 °C. Fatty acids were identified by comparing retention times with those of authentic standards (Supelco 37 FAME C4-C24) using Agilent ChemStation B.03.01 software. Fatty acid concentrations were expressed as μg g^−1^ wet mass of tissue.

To evaluate the nutritional quality of the fatty acids in terms of their potential for preventing cardiovascular disease, the atherogenic (AI), thrombogenic (TI), and hypocholesterolemic/hypercholesterolemic (h/H) indices were calculated, following the formulas described by Chen and Liu [49]:

(1) $$AI=\frac{C12:0+4\cdot C14:0+C16:0}{\sum MUFA+\sum n3+\sum n6}$$

(2) $$TI=\frac{C14:0+C16:0+C18:0}{0.5\cdot \sum MUFA+0.5\cdot \sum n6+3\cdot \sum n3+\frac{\sum n3}{\sum n6}}$$

(3) $$h/H=\frac{C18:1+C18:2+C18:3+C20:1+C22:1+C24:1}{C14:0+C16:0}$$

where ∑*MUFA*—total monounsaturated fatty acids, ∑*PUFA*—total polyunsaturated fatty acids, *n3*—omega-3 PUFA, and *n6*—omega-6 PUFA.

### 2.3. Data Analysis

Carapace lengths and weights in male and female crabs were compared using a non-parametric Kruskal–Wallis test because the data had non-normal distribution. To visualize the fatty acid patterns in red king crabs, Principal Component Analysis (PCA) was used. Initially, the fatty acid data were compared between males and females using a multivariate PERMANOVA based on the Bray–Curtis dissimilarity index with 9999 permutations. Differences in individual fatty acid concentrations were assessed using the Kruskal–Wallis test (KWT), as the data did not pass the Shapiro–Wilk test for normal distribution for most fatty acids. Based on the results obtained from the preliminary tests, the fatty acid profiles were then compared for males and females separately using both PERMANOVA and the paired samples Wilcoxon test for non-normally distributed data. To address the increased Type I error risk from multiple comparisons, false discovery rate correction was applied to *p*-values from the Wilcoxon tests.

Data are presented as means ± SE (standard error).

## 3. Results

**Figure 1:**
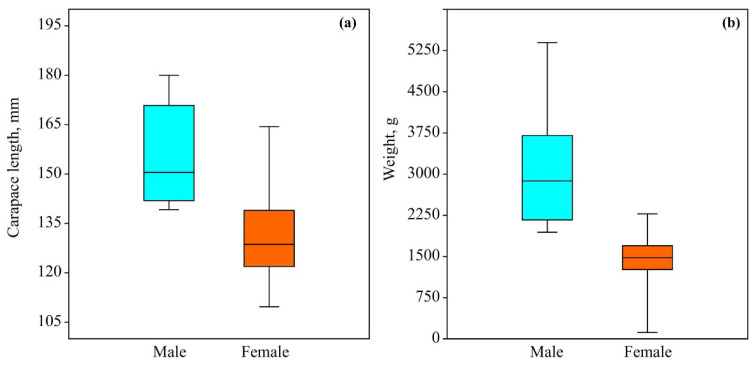
Mean carapace length (**a**) and weight (**b**) of red king crabs collected in Dalnezelenetskaya Bay, Barents Sea, in summer 2021.

Our sample size included 84 red king crabs: 16 males and 68 females. Male crabs attained a larger carapace length (df = 1, H = 26.81, *p* < 0.001) and were heavier (df = 1, H = 35.92, *p* < 0.001) than female crabs (Figure 1).

A total of 43 fatty acids were detected in the raw and cooked meat of the red king crab (Table 1 and Table 2), with palmitic (C16:0) and stearic acids (C18:0) being the most prevalent saturated fatty acids (SFA), oleic (C18:1n9C) and palmitoleic acids (C16:1C) dominating among monounsaturated fatty acids, and eicosapentaenoic acid (EPA, C20:5n3), docosahexaenoic acid (DHA, C22:6n3), and arachidonic acid (C20:4n6) prevailing among polyunsaturated fatty acids (PUFA).

**Figure 2:**
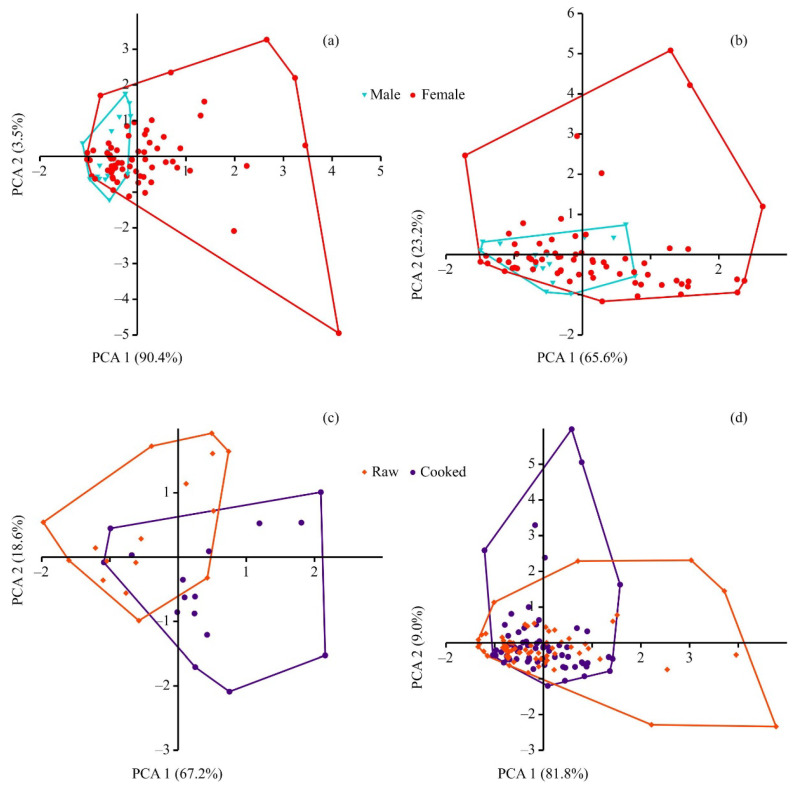
PCA plots based on a Bray–Curtis similarity matrix for fatty acid profiles of red king crab meat in Dalnezelenetskaya Bay, Barents Sea, in summer 2021. (**a**) Raw meat, male vs. female; (**b**) cooked meat, male vs. female; (**c**) male, raw vs. cooked meat; (**d**) female, raw vs. cooked meat.

The PCA applied to the data describing the fatty acid content in raw meat showed a separation between male and female crabs (Figure 2a).

The first axis explained 90.4% of the total variation and was positively scaled with most fatty acids; the highest correlations were found for C20:5n3, C16:0, and C18:1n9C. Despite the overlapping distributions of male and female profiles in the left side of the plot, approximately 46% of female profiles had positive PCA scores (right side), which resulted in significant differences between males and females (PERMANOVA, df = 1, H = 6.85, *p* = 0.005). According to the Bray–Curtis coefficient, the degree of dissimilarity between males and females was 23.64%. In the case of cooked meat, PCA also indicated a separation between males and females, with Axes 1 and 2 accounting for 65.56% and 23.23%, respectively (Figure 2b). The content of C20:5n3 had the highest correlation with both axes, but it was positive for Axis 1 and negative for Axis 2. There was a notable overlap in the distribution of fatty acid data, leading to an insignificant difference between males and females (PERMANOVA, df = 1, F = 2.90, *p* = 0.056) at a dissimilarity level of 17.64%. Comparisons conducted for individual fatty acids confirm the results of the PCA. For raw meat, 21 of the 43 fatty acids found in crabmeat showed significant differences in content, with higher values being registered in females, which also resulted in a significantly higher total content in females (6323 ± 375 vs. 4339 ± 249 μg g^−1^). For cooked meat, the total content was also higher in females but with no significant differences (5920 ± 197 vs. 5157 ± 291 μg g^−1^), and the number of significantly different individual fatty acids was 10.

The PCA conducted for male crabs separated the data for raw and cooked meat mainly along the first axis, which explained 67.18% of the variation (Figure 2c) and had the highest positive correlations with C20:5n3, C16:0, C22:6n3, and C18:1n9C. The second axis explained 18.64% of the total variation and had the highest positive correlations with C20:4n6, C16:0, C18:0, and C18:1n9C. Thus, most profiles of raw meat had negative PC1 and PC2 scores, while cooked meat profiles had positive scores. PERMANOVA showed significant differences (df = 1, F = 4.10, *p* = 0.021) with a dissimilarity level of 17.64%. Paired Wilcoxon tests indicated significant differences for four fatty acids, including eicosapentaenoic acid, with a higher content in cooked meat. SFA and MUFA contents were similar, whereas polyunsaturated fatty acid (PUFA) and omega-3 (but not omega-6) contents were higher in the cooked meat (Table 1).

In the case of female crabs, PCA showed that the first axis accounted for 81.76% of the total variation (Figure 2d) and positively correlated with EPA content, while the second axis, explaining 8.98% of the variation, was negatively scaled with EPA content. Most stations overlapped, which resulted in a dissimilarity level of 22.03% between raw and cooked meat. Nevertheless, differences between the fatty acid profiles were significant (PERMANOVA, df = 1, F = 4.09, *p* = 0.022). Further analysis indicated significant differences with regard to 18 fatty acids (Table 2), with higher values in raw meat detected for some prevalent fatty acids, including C18:0, C20:1n9, C20:2n6, and C20:4n6. At the same time, the contents of SFA, MUFA, and PUFA, as well as the total content, were similar in raw and cooked meat derived from females. Significant differences were detected for the omega-6 content (Table 2).

Nutrition indices did not differ significantly between males and females for either raw or cooked meat (*p* > 0.05). The n-3/n-6 ratio was also similar in males and females. Further comparisons showed significant differences between raw and cooked meat derived from males (Table 1) in the case of the TI (0.19 vs. 0.16) and the PUFA/SFA ratio (1.89 vs. 2.04). Regarding females, significant differences were found for the AI (raw vs. cooked meat, 0.29 vs. 0.34), the h/H ratio (4.01 vs. 3.40), the PUFA/SFA ratio (2.03 vs. 1.88), and the n-3/n-6 ratio (3.57 vs. 4.80) (Table 2).

## 4. Discussion

We found that male red king crabs had greater size and weight compared to female crabs, reflecting sexual dimorphism in this species. Males allocate more energy towards somatic growth, while females invest more energy in gonad growth, egg development, and protection of egg masses during the extended 11.5-month embryogenesis and larval release period [19,35].

Previous studies conducted in Norwegian waters have reported that the contents of omega-6, omega-3, PUFA, EPA + DHA, and total fatty acids in cooked red king crab male meat were 500, 1930, 2430, 1870, and 4800 μg g^−1^, respectively [42]. These levels were lower than those found in the present study, which may be attributed to differences in the feeding habits of the crabs [29,30,50], seasonal variations in crab physiology, and/or variations in the thermal regimes of the coastal waters of Norway [51] and Russia [52].

Our biochemical assays revealed significant differences in fatty acid profiles between male and female crabs. This result may be attributed to variations in the feeding habits of males and females. Most male crabs appear to migrate from deeper water locations to the shallow water area of Dalnezelenetskaya Bay, possibly seeking more favorable feeding conditions [19]. In contrast, stationary female crabs residing in the coastal sites have the opportunity to accumulate more fatty acids in their organs and meat. The recent warming trend in the coastal Barents Sea [15,53] is responsible for increased production and biomass of benthic animals [18], which serve as food sources for red king crabs [29,30]. Furthermore, male crabs have larger legs than females [35], which reflects not only their higher energy allocation to growth but also the heavier load and more frequent use of leg muscles during migration. This may contribute to lower fat content and, consequently, lower fatty acid levels in male muscle tissues. Significant sex-based variations in the biochemical composition, including fatty acid profiles, have been reported in various crab species from different regions, such as the long-eyed swimming crab *Podophthalmus vigil* from India [54], the blue crab *Callinectes sapidus* from the eastern Mediterranean Sea [55,56], the swimming crab *Portunus segnis* from Tunisia [57], the Chinese mitten crab *Eriocheir sinensis* from China [58] and Poland [59], and the warty crab *Eriphia verrucosa* from the southeastern Black Sea [60]. In most cases, the female edible parts have been found to contain higher contents of fatty acids compared to the males, which is consistent with the findings of the present study.

We observed a predominance of PUFA in the red king crab meat samples, followed by SFA and MUFA. This pattern, characterized by higher PUFA content, aligns with findings in other shellfish species [61,62], although the specific fatty acid levels can vary significantly between species. For instance, our measurements of these fatty acid families for the raw meat of female red king crabs were 3431, 1706, and 1186 μg g^−1^, which are higher than those found in the raw meat of the Atlantic spider crab, *Maja brachydactyla*, from Portugal (1050, 420, 470 μg g^−1^, [63]) and the blue swimming crab, *Portunus segnis*, from Tunisia (329, 300, 206 μg g^−1^, [57]).

Male red king crabs exhibited lower levels of PUFA, SFA, and MUFA in their raw meat (2253, 1254, and 832 μg g^−1^) compared to the European edible crab, *Cancer pagurus*, from the Scottish coast (19,320, 18,360, and 30,340 μg g^−1^, [64]) and the coconut crab, *Birgus latro*, from Okinawa, southwest Japan (415,700, 244,000, and 266,200 μg g^−1^, [65]). Additionally, the raw and cooked meat of male red king crabs contained lower amounts of EPA, DHA, palmitic, and oleic acids (raw meat: 1125, 575, 737, and 598 μg g^−1^; cooked meat: 1486, 694, 856, and 696 μg g^−1^) compared to southern king crabs, *Lithodes santolla*, from the southwestern Atlantic, San Jorge Gulf (raw meat: 1200, 774, 1110, and 1530 μg g^−1^; cooked meat: 1660, 1030, 1170, and 1680 μg g^−1^; [66]). Conversely, the raw meat of male red king crabs had similar contents of PUFA, SFA, and MUFA to those found in the grey swimming crab, *Liocarcinus vernalis*, from the central Adriatic Sea (2916, 1185, and 1138 μg g^−1^, [67]), but exceeded the levels reported for the Chinese mitten crab, *Eriocheir sinensis*, from the Odra Estuary (1431, 961, and 1208 μg g^−1^, [59]). These variations in fatty acid profiles can be attributed to the different physiologies of these crab species, which reflect their adaptations to distinct habitat conditions [68,69] and food preferences [70,71,72]. Our study revealed significant differences in the fatty acid profiles between raw and cooked meat, with these differences being more pronounced in males. This finding likely reflects differences in muscle structure between the more active male crabs and the more stationary female red king crabs [19,73]. We observed increases in the contents of important fatty acids in males after cooking, including EPA (32%), DHA (21%), PUFA (24%), and total fatty acids (19%). Similar findings have been reported for other crab species. For example, cooked claw meat of *Carcinus maenas* contained 29% higher EPA and 37% higher DHA levels than raw meat [74]. In *Lithodes santolla*, higher increases of 38% and 33% for EPA and DHA, respectively, were found in cooked meat compared to raw meat [66]. These increases in fatty acid concentrations can be attributed to multiple interconnected mechanisms. First, the heating process induces substantial water loss from muscle tissue, concentrating the absolute quantity of fatty acids in the remaining dry matter without necessarily increasing their total mass [75]. Second, thermal denaturation of muscle proteins disrupts the protein matrix, simultaneously releasing occluded lipids and reducing protein mass, thereby increasing the proportional contribution of fatty acids to total tissue composition [76]. Third, the leaching of water-soluble components, including amino acids, nucleotides, and carbohydrates, further concentrates the lipid fraction relative to other tissue constituents. Additional increases in polyunsaturated fatty acid content may result from enhanced extraction of phospholipids during thermal processing. Phospholipids, which preferentially contain long-chain PUFAs at the sn-2 position of the glycerol backbone [77], become more accessible to analytical extraction procedures following protein denaturation. However, this pattern of increased fatty acid concentrations is not universal across crustacean species or cooking conditions. Notably, boiled meat of *Cancer pagurus* demonstrated substantially lower fatty acid contents than raw meat, with reductions of 31% for SFA and PUFA, and 45% for MUFA and DHA [64]. This divergent response is likely attributable to the higher cooking temperature employed (105 °C versus 95 °C in our study), which promotes oxidative degradation and loss of thermolabile long-chain fatty acids [66].

Omega-3 and omega-6 fatty acids are not interconvertible in the human body [78] and are important components of practically all cell membranes [79,80]. While cellular proteins are genetically determined, the PUFA composition of cell membranes is, to a great extent, dependent on dietary intake [81]. The fat in land animal meats, such as beef, lamb, and pork, as well as some tropical oils, like coconut oil, contain high levels of saturated fat [82]. In contrast, fatty fish and shellfish are rich sources of the “good” omega-3 fats [83,84].

Increasing evidence from animal and in vitro studies indicates that the long-chain omega-3 PUFAs, especially EPA and DHA, present in fatty fish, fish oils, and shellfish products can reduce the risk of cardiovascular diseases and inhibit carcinogenesis [85,86,87]. Furthermore, the consumption of seafood products and by-products containing PUFAs has been shown to have other positive effects on human health, such as antidepressant [88], anti-diabetic [85], antihyperglycemic [89], antimicrobial [90], antioxidant [91], anti-rheumatic [92], and immunomodulatory properties [93]. However, different marine products can vary considerably in terms of dietary benefits due to different proportions of SFA, MUFA, and PUFA. To evaluate the quality of these products, nutritional indices have been widely used. In particular, a PUFA/SFA ratio exceeding 0.45, an n-3/n-6 ratio higher than 4, an AI lower than 1, and a TI lower than 0.5 are considered indicators of high-quality products. Additionally, higher estimates for the h/H index are deemed better than lower values [49], and an ideal n-3/n-6 ratio is established at 4:1 [94].

**Figure 3:**
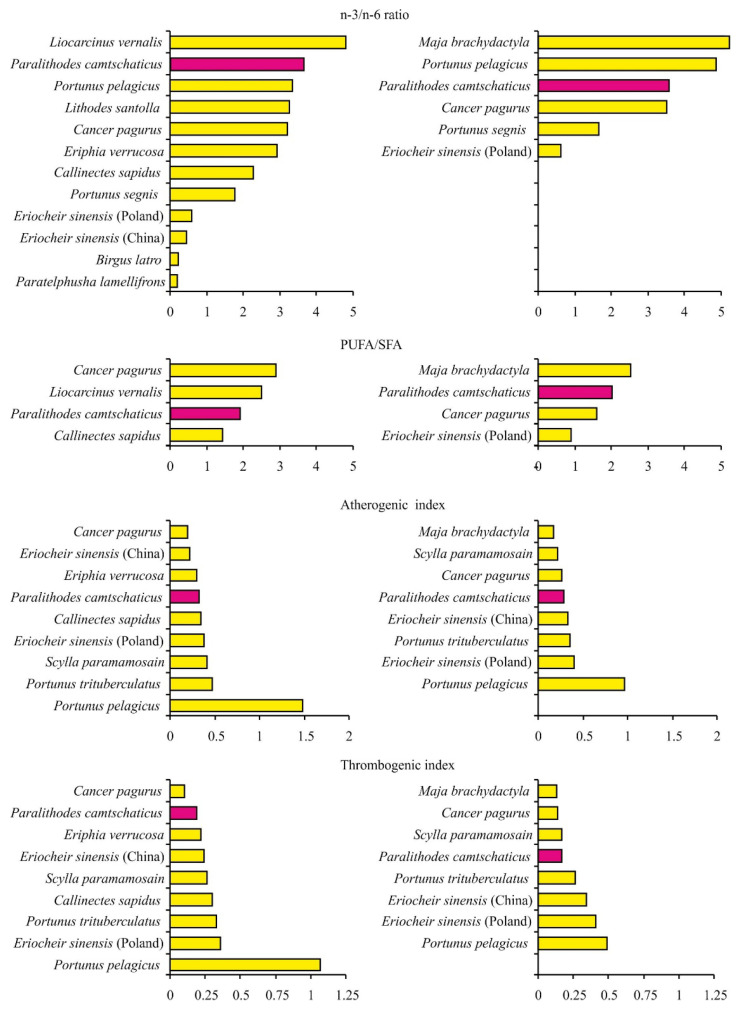
Variations in nutritional indices calculated for raw meat of different crab species [56,57,59,63,65,66,67,70,71,95,96,97,98,99,100]. Left side—males; right side—females.

The calculated nutrition indices of red king crab meat are within the limits established for healthy diets. Moreover, these indices are often more beneficial than those reported for other crab species, predominantly those inhabiting warmer locations, such as *Callinectes sapidus*, *Eriocheir sinensis*, *Portunus pelagicus*, and *Scylla paramamosain*, due to the higher levels of PUFAs in red king crabs (Figure 3).

This is likely due to the adaptations of poikilothermic organisms to colder environments, which involve modulating the lipid composition of their membranes to maintain a relatively constant membrane fluidity, including alterations in the degree of fatty acid unsaturation, chain length, and the proportion of branched-chain fatty acids [101].

Thus, the high concentrations of DHA and EPA, coupled with an n-3/n-6 ratio close to the recommended value, highlight the nutritional importance of red king crab meat. It plays a significant role in human diets and can help prevent cardiovascular diseases and undesirable inflammatory processes.

## 5. Conclusions

Our study provides novel insights into the fatty acid composition of the meat derived from red king crabs inhabiting the Barents Sea. We found that the fatty acid content in the meat from males is lower than that recorded in females, probably due to differences in the physiology, behavior, and/or activity between males and females. The cooking procedure has different effects on the meat, depending on crab sex. There is a significant improvement recorded for male meat, and less expressed, but still significant, changes for female meat, which also likely reflects differences in the tissue structure between males and females. Despite the lower overall fatty acid levels in red king crab meat compared to other crabs, this product in many cases has a more balanced ratio of PUFA to SFA contents, resulting in better nutritional indices, making the red king crab meat an excellent product for a healthy diet. Further research is needed to reveal the underlying mechanisms for the patterns established in the current study.

## Figures and Tables

**Table 1:** Fatty acid composition in raw and cooked meat of male red king crabs (μg g^−1^) from Dalnezelenetskaya Bay, Barents Sea, in summer 2021.

Fatty Acid	Raw			Cooked			Comparison	
	X ± SE	Min	Max	X ± SE	Min	Max	z	*p*
C6:0	4 ± 0.7	0.7	12.2	5.3 ± 1.1	1.3	19.9	1.19	0.775
C8:0	7.9 ± 1.4	0.5	16.7	8.2 ± 1.3	1.9	18.9	0.16	0.986
C9:0	3.6 ± 0.8	1.0	13.2	3.2 ± 0.5	0.7	9.2	0.41	0.986
C10:0	3.1 ± 0.6	1.3	11	3 ± 0.4	1.3	5.5	0.88	0.859
C11:0	1.6 ± 0.2	0.8	4.8	1.3 ± 0.1	0.6	1.9	1.40	0.699
C12:0	24.8 ± 6	7.7	109	19.5 ± 2.3	8.8	44.7	0.21	0.986
C13:0	1.5 ± 0.3	0.4	4.1	1.4 ± 0.1	0.4	2.7	0.26	0.986
C14:0	56.8 ± 6	25.4	117	63.4 ± 5.8	33.9	116	1.65	0.699
C15:0	37.3 ± 3.4	15.7	63.2	43.7 ± 2.7	31.8	63.7	2.07	0.091
C16:0	737 ± 49.5	403	1035	856 ± 51	652	1302	1.86	0.699
C17:0	39.2 ± 3.2	18.6	65.4	45.1 ± 2.8	28.3	68.1	1.71	0.699
C18:0	313 ± 32.5	187	617	305 ± 22.3	193	505	0.26	0.986
C20:0	12.3 ± 1.7	4.2	29.6	10.4 ± 1.3	5.1	22	0.52	0.964
C21:0	7.4 ± 0.7	4.7	14.1	8.2 ± 1.1	2.6	18.5	0.62	0.964
C22:0	1.6 ± 0.4	0.8	4.9	1.6 ± 0.2	0.3	3.0	0.27	0.986
C23:0	1.5 ± 0.3	0.6	5.0	1.3 ± 0.2	0.4	3.9	0.78	0.942
C24:0	5.1 ± 0.8	2.5	14.8	4.9 ± 0.5	2.1	11.2	0.52	0.964
C14:1n5t	0.6 ± 0.2	0.2	1.6	0.3 ± 0.1	0.1	0.7	1.36	0.699
C14:1n5C	2 ± 0.4	0.4	8.1	2.2 ± 0.4	0.9	6.8	0.31	0.986
C15:1n5	0.6 ± 0.1	0.2	1.2	0.3 ± 0.1	0.2	0.6	0.94	0.859
C16:1n7t	1 ± 0.3	0.1	4.7	0.9 ± 0.1	0.3	2.4	0.60	0.964
C16:1n7C	146 ± 18.9	46.3	300	197 ± 25.8	95.5	396	2.53	**0.047**
C17:1n7	1.5 ± 0.2	0.5	3.3	2.1 ± 0.1	1.2	3.2	2.28	0.072
C18:1n9t	24.5 ± 4.4	6.4	79.3	30.8 ± 7.9	13.1	144	0.52	0.964
C18:1n9C	598 ± 46	271	812	696 ± 40.4	444	1010	1.34	0.699
C20:1n9	52.9 ± 6.9	18.8	117	53 ± 8.7	18.9	135	0.31	0.986
C22:1n9	5.3 ± 0.6	1.5	10.6	4.2 ± 0.5	0.8	7.9	1.29	0.703
C24:1n9	0.7 ± 0.1	0.2	1.6	1 ± 0.2	0.4	3.3	2.04	0.091
C18:2n6t	6.6 ± 1	1.7	10.9	6.9 ± 1.1	1.1	11.3	0.56	0.964
C18:2n6C	56.8 ± 4.1	28.9	96.5	73.6 ± 4.4	47.2	103	2.43	**0.049**
C18:3n3	15.6 ± 1.6	3.2	31.4	21.8 ± 2.4	8.8	48.4	3.15	**0.016**
C18:3n6	1.1 ± 0.3	0.3	4.9	1 ± 0.1	0.3	2.4	0.74	0.943
C20:2n6	41 ± 3.2	19.7	59.8	44.8 ± 4.5	23.4	77.5	0.93	0.859
C20:3n6	6.2 ± 0.8	2.6	12.1	6.3 ± 0.8	2.9	14.3	0.00	1.000
C20:4n6	395 ± 45.4	108	748	373 ± 31.5	213	615	0.31	0.986
C22:2n6	0.6 ± 0.1	0.2	1.7	0.6 ± 0.1	0.3	1.1	0.05	1.000
C20:5n3	1125 ± 45.8	714	1495	1486 ± 72.4	961	2110	3.31	**0.016**
C22:6n3	575 ± 46.6	218	951	694 ± 50	374	1072	1.87	0.699
C20:3n3	7.2 ± 0.8	2.2	12.1	8.4 ± 1	3.3	18	1.14	0.784
C22:4n6	8.2 ± 1.6	3.0	24.7	10 ± 2.1	1.7	29.6	0.16	0.986
C22:3n3	0.5 ± 0.1	0.2	1.5	0.5 ± 0.1	0.2	1.4	0.89	0.859
C22:5n6	12.3 ± 1.7	2.9	27.2	12.8 ± 1.9	4.6	29.5	0.10	0.986
C22:5n3	40 ± 6.3	9.7	102	52 ± 9.1	12.3	118	0.98	0.859
SFA	1254 ± 93.9	696	2084	1380 ± 83.7	1043	2086	1.34	0.699
MUFA	832 ± 63.3	467	1199	988 ± 70.1	677	1586	1.55	0.699
PUFA	2253 ± 122	1168	3178	2789 ± 153	1838	3801	2.53	**0.047**
Total	4339 ± 249	2793	5802	5157 ± 291	3559	7380	2.02	0.091
AI	0.32 ± 0.02	0.22	0.58	0.3 ± 0.01	0.23	0.37	0.16	0.986
TI	0.19 ± 0.02	0.12	0.37	0.16 ± 0.01	0.12	0.19	2.17	0.083
h/H	3.65 ± 0.22	1.81	5.03	3.77 ± 0.15	3.1	4.83	0.10	0.986
PUFA/SFA	1.89 ± 0.11	1.01	2.58	2.04 ± 0.08	1.69	2.67	1.96	0.095
n-3	1727 ± 88.4	960	2267	2263 ± 118	1520	3156	3.10	**0.016**
n-6	526 ± 52.1	208	911	526 ± 43	318	850	0.00	1.000
n-9	681 ± 51.8	388	936	785 ± 49.4	505	1183	1.34	0.699
n-7	148 ± 18.9	47	302	200 ± 25.9	99.2	399	2.53	**0.047**
n-3/n-6	3.65 ± 0.28	1.6	5.59	4.48 ± 0.2	2.94	5.54	1.86	0.699

Note. X—mean, SE—standard error, Min—minimum, Max—maximum, SFA—saturated fatty acids, MUFA—monounsaturated fatty acids, PUFA—polyunsaturated fatty acids, n-3—omega-3 fatty acids, n-6—omega-6 fatty acids, n-9—omega-9 fatty acids, n-7—omega-7 fatty acids, AI—atherogenic index, TI—thrombogenic index, h/H—ratio between hypocholesterolemic and hypercholesterolemic fatty acids, z—statistics for paired Wilcoxon tests, *p*—probability level (adjusted). Bold font denotes significant statistical differences for fatty acid contents between raw and cooked meat (*p* < 0.05).

**Table 2:** Fatty acid composition in raw and cooked meat of female red king crabs (μg g^−1^) from Dalnezelenetskaya Bay, Barents Sea, in summer 2021.

Fatty Acid	Raw			Cooked			Comparison	
	X ± SE	Min	Max	X ± SE	Min	Max	z	*p*
C6:0	3.8 ± 0.2	1.0	11.3	4.1 ± 0.4	1.0	19.9	0.09	0.980
C8:0	5.3 ± 0.6	0.7	18.3	6 ± 0.7	0.6	26.4	1.13	0.792
C9:0	2.9 ± 0.3	1.0	15.9	3.2 ± 0.3	0.9	11.2	0.92	0.792
C10:0	2.6 ± 0.2	0.7	10.4	2.2 ± 0.2	0.7	6.4	1.95	0.065
C11:0	1.3 ± 0.1	0.4	3.9	1.6 ± 0.2	0.4	7.7	1.17	0.244
C12:0	22.6 ± 2.2	3.9	96.7	17.6 ± 1.2	5.0	42.6	2.33	**0.030**
C13:0	1.7 ± 0.1	0.7	6.1	1.9 ± 0.1	0.7	5.7	0.97	0.792
C14:0	82.9 ± 5	27.9	217	80.8 ± 3.8	42	195	0.14	0.980
C15:0	45.1 ± 2.3	23.2	109	50.8 ± 2.2	26	123	2.06	0.052
C16:0	978 ± 58.9	452	2659	1088 ± 47.1	575	2775	2.13	**0.048**
C17:0	60.5 ± 3.8	28.3	237	59 ± 2.4	27.9	142	0.06	0.980
C18:0	452 ± 26.3	170	1084	361 ± 16.4	202	976	3.33	**0.002**
C20:0	21.3 ± 1.4	6.7	68.4	13.2 ± 0.9	5.2	37.2	5.49	**0.000**
C21:0	18.6 ± 1.2	6.8	53.7	14.1 ± 0.7	6.1	36	3.92	**0.000**
C22:0	2.1 ± 0.3	1.3	3.1	1.3 ± 0.3	0.3	3.2	1.21	0.232
C23:0	1.9 ± 0.1	0.6	5.3	1.3 ± 0.1	0.4	3.3	4.87	**0.000**
C24:0	5.7 ± 0.3	1.7	16.3	4.8 ± 0.2	0.7	10.3	2.37	**0.028**
C14:1n5t	0.5 ± 0.1	0.2	1.6	0.6 ± 0.1	0.1	1.5	0.86	0.792
C14:1n5C	2.8 ± 0.3	0.2	14.4	2.2 ± 0.1	0.2	6.3	2.64	**0.015**
C15:1n5	0.3 ± 0.1	0.1	0.8	0.5 ± 0.1	0.2	1.2	0.94	0.792
C16:1n7t	0.8 ± 0.1	0.1	4.5	0.9 ± 0.1	0.2	3.1	0.82	0.792
C16:1n7C	205 ± 18	62.2	714	217 ± 15	94.9	784	1.57	0.138
C17:1n7	1.9 ± 0.1	0.4	4.9	2.4 ± 0.2	0.6	9.3	2.07	0.052
C18:1n9t	26.7 ± 2.3	1.9	96.1	25.9 ± 3.1	3.9	147	1.08	0.792
C18:1n9C	854 ± 54.9	368	2434	803 ± 31.8	402	1520	0.32	0.980
C20:1n9	86.1 ± 7.1	19.9	290	52.9 ± 3.7	19.3	172	5.17	**0.000**
C22:1n9	9.1 ± 0.7	2.4	35.5	5.3 ± 0.3	0.8	13.8	5.14	**0.000**
C24:1n9	0.6 ± 0.1	0.2	1.3	1 ± 0.3	0.3	2.9	1.15	0.792
C18:2n6t	6.1 ± 1.1	2.1	15.3	5.2 ± 1.3	0.9	14.8	0.14	0.980
C18:2n6C	80.6 ± 6.4	34.5	302	94 ± 4.6	38	223	2.76	**0.011**
C18:3n3	30.3 ± 2.6	9.0	96.7	31.4 ± 1.8	12.2	84.2	1.25	0.225
C18:3n6	1.3 ± 0.2	0.2	6	1.2 ± 0.1	0.2	5.8	0.62	0.949
C20:2n6	67.5 ± 4.3	23.9	223	56.1 ± 2.5	27	135	2.52	**0.019**
C20:3n6	8.6 ± 0.8	2.2	42	5.7 ± 0.3	2.4	14.8	4.55	**0.000**
C20:4n6	601 ± 41.3	146	1978	390 ± 21.8	142	1031	5.03	**0.000**
C22:2n6	1.1 ± 0.1	0.3	3.2	0.7 ± 0.1	0.1	2.6	5.20	**0.000**
C20:5n3	1673 ± 103	705	5526	1689 ± 59.6	233	2856	1.12	0.792
C22:6n3	858 ± 50	282	2200	758 ± 28.9	55.7	1228	1.42	0.172
C20:3n3	16.9 ± 1.6	4.4	85.7	14.7 ± 1.1	5.9	48.7	1.52	0.145
C22:4n6	12.6 ± 1.1	2.8	57	7.4 ± 0.6	2.5	26.9	5.38	**0.000**
C22:3n3	0.5 ± 0	0.2	1.6	0.5 ± 0	0.2	0.9	0.84	0.792
C22:5n6	21.9 ± 1.9	4.6	92	13.8 ± 1.2	3.8	71.2	5.00	**0.000**
C22:5n3	57.6 ± 4.2	14.5	151	38.2 ± 2.3	15.9	110	4.77	**0.000**
SFA	1706 ± 98.1	753	4388	1709 ± 72	940	4249	0.34	0.980
MUFA	1186 ± 79.7	504	3352	1110 ± 47.7	547	2575	0.29	0.980
PUFA	3431 ± 202	1416	9721	3101 ± 106	1440	5160	1.11	0.792
Total	6323 ± 375	2906	17006	5920 ± 197	3380	10583	0.40	0.980
AI	0.29 ± 0.01	0.16	0.43	0.34 ± 0.01	0.26	0.62	5.72	**0.000**
TI	0.17 ± 0	0.11	0.29	0.19 ± 0.01	0.13	0.52	0.02	0.980
h/H	4.01 ± 0.08	2.38	6.91	3.4 ± 0.07	1.38	4.43	6.10	**0.000**
PUFA/SFA	2.02 ± 0.03	1.23	2.87	1.88 ± 0.04	0.69	2.43	2.88	**0.008**
n-3	2636 ± 155	1149	7124	2532 ± 86.8	898	4096	0.07	0.980
n-6	796 ± 53.2	236	2597	570 ± 28	238	1418	4.24	**0.000**
n-9	975 ± 63.1	414	2684	887 ± 35.9	443	1801	0.91	0.792
n-7	208 ± 18.1	63.9	717	220 ± 15.1	97.7	790	1.58	0.138
n-3/n-6	3.57 ± 0.14	1.81	7.93	4.8 ± 0.17	1.65	9.36	5.63	**0.000**

Note. X—mean, SE—standard error, Min—minimum, Max—maximum, SFA—saturated fatty acid, MUFA—monounsaturated fatty acid, PUFA—polyunsaturated fatty acid, n-3—omega-3 fatty acid, n-6—omega-6 fatty acid, n-9—omega-9 fatty acid, n-7—omega-7 fatty acid, AI—atherogenic index, TI—thrombogenic index, h/H—ratio between hypocholesterolemic and hypercholesterolemic fatty acids, z—statistics for paired Wilcoxon tests, *p*—probability level (adjusted). Bold font denotes significant statistical differences for fatty acid contents between raw and cooked meat (*p* < 0.05).

## Data Availability

The original contributions presented in this study are included in the article. Further inquiries can be directed to the corresponding authors.
